# Effects of animal fat replacement with almond flour on quality parameters of beef patties

**DOI:** 10.1002/fsn3.3633

**Published:** 2023-08-23

**Authors:** Mine Kirkyol, Ahmet Akköse

**Affiliations:** ^1^ Faculty of Agriculture, Department of Food Engineering Atatürk University Erzurum Turkey

**Keywords:** almond flour, beef patty, fatty acid composition, hardness, TBARS, texture

## Abstract

Almond is rich in vitamins, minerals, and dietary fiber and contains high fat and protein. For this reason, almond flour can be used as an additive in the production of various foods to increase nutritional value, improve texture and flavor, and produce healthier products. The purpose of this study is to determine the availability of almond flour as an animal fat replacer in the production of beef patties. For this purpose, beef patties were produced in five different formulations containing animal fat and/or almond flour, and pH, moisture content, color, and TBARS values were detected in both raw and cooked samples. In addition, cooking yield and shrinkage were calculated and fatty acid composition, texture profile, and sensory analyses were performed on cooked samples. Replacing animal fat with almond flour increased pH in raw and cooked beef patties but decreased moisture content, b* value, and TBARS in cooked samples compared to the control. While cooking yield increased in beef patties containing almond flour, shrinkage decreased. In addition, the cooking process caused decreases in L*, a*, and b* values. Using almond flour in beef patties decreased the SFAs and increased the amounts of oleic and linoleic acids. Hardness, cohesiveness, resilience, and chewiness were significantly affected by the use of almond flour (*p* < .01), and higher hardness and chewiness, and lower cohesiveness and resilience were determined in the samples containing almond flour compared to the control. On the other hand, the use of almond flour instead of animal fat in beef patties did not have a significant effect on the determined sensory properties (*p* > .05).

## Graphical abstract

Consumers' interest in nutrition and health has led the meat and meat product industry to produce functional products, as in other branches of the food industry. In this study, almond flour was used as a fat replacer in beef patties to obtain a healthier meat product, and the physicochemical, textural, and sensory properties of beef patties were investigated. It was concluded that almond flour could be used in beef patties as an animal fat replacer.

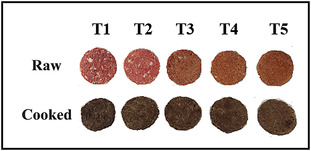

## 1 INTRODUCTION

Meat, an important source of protein, is an essential food that has an important place in human nutrition due to its B‐group vitamins and mineral substances such as iron and zinc. Fresh meat can be marketed as pieces or minced meat, or it can be processed into various products and offered for consumption. Beef patty, one of these products, is a meat product obtained by mixing minced meat, animal fat, and flavorings with some other additives and shaping this mixture into small portions. In this context, beef patty can be prepared and consumed in different compositions and forms throughout the world (Aslinah et al., 2018).

Animal fats in the composition of meat products play an important role in the formation of structural and sensory properties. It is reported that animal fats in meat products affect flavor, texture, juiciness, and desired mouth feel (Choi et al., 2016). Moreover, fat is an important source of energy and essential fatty acids and is a carrier of fat‐soluble vitamins (Choi et al., 2009; Vural et al., 2004). However, since animal fats contain high amounts of saturated fatty acids and cholesterol, they are associated with health problems such as obesity, hypertension, and cardiovascular diseases (Choi et al., 2016). In this context, consumers' interest in foods with reduced fat and high fiber content is increasing (El Zeny et al., 2019; Modi et al., 2009). For this reason, the number of studies on reducing or replacing animal fat in meat products is increasing day by day (Jo et al., 2003; Yılmaz & Dağlıoğlu, 2003; Serdaroglu & Degirmencioglu, 2004; Turhan et al., 2005; Pelser et al., 2007; Turhan et al., 2007; Valencia et al., 2008; Bilek & Turhan, 2009; Bastos et al., 2014; Choi et al., 2016; Rabadan et al., 2021; Mumyapan et al., 2021).

Containing high protein, fat, and dietary fiber, almonds are an important source of vitamin E, riboflavin, and essential minerals (Kendall et al., 2010; Yada et al., 2013). It is also considered a low glycemic index food due to its low carbohydrate and high unsaturated fat content (Martins et al., 2017). A significant portion of the oil in almonds consists of mono and polyunsaturated fatty acids (90%), among which the oleic acid content (70%) stands out (Karatay et al., 2014; Kırbaşlar et al., 2012; Matthäus et al., 2018). It has been reported that the consumption of almonds reduces the amount of low‐density lipoprotein (LDL), thus providing various health benefits such as reduction in coronary heart disease, cholesterol, hypertension, diabetes, obesity, and oxidative stress (Ahrens et al., 2005; Chen et al., 2017; Hou et al., 2018; Kamil & Chen, 2012; Richardson et al., 2009; Zacheo et al., 2000). In addition to consumption as a snack, almonds can also be ground into flour and used as an additive in the production of foods. Studies on the use of almond flour in foods have generally focused on bakery products, and there are limited studies on its use in some other foods such as milk and meat products. (Akesowan, 2015; Hopkin et al., 2022; Jabeen et al., 2022; Mazzaglia et al., 2020; Rabadan et al., 2021; Yildiz & Gocmen, 2021). However, there is no study in the literature in which almond flour is used as a fat replacer in the production of beef patties. The purpose of this study is to evaluate the potential of almond flour as a fat replacer in the production of beef patties. For this purpose, animal fat was substituted with almond flour in beef patty production, and the effects on physicochemical, textural, and sensory properties were investigated.

## 2 MATERIALS AND METHODS

### 2.1 Beef patty production

**FIGURE 1:**
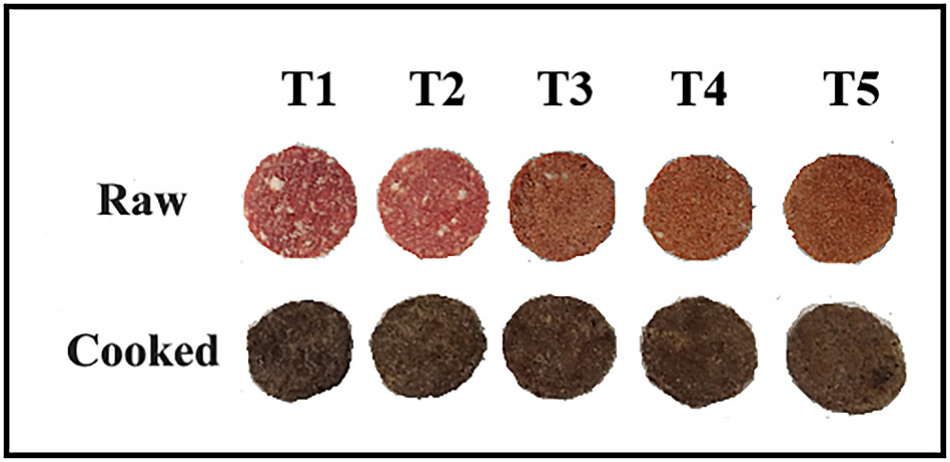
Physical appearance of raw and cooked beef patties produced in different formulations (T1: 100% animal fat, T2: 75% animal fat + 25% almond flour, T3: 50% animal fat + 50% almond flour, T4: 25% animal fat + 75% almond flour, T5: 100% almond flour).

In the study, beef meat, beef fat, NaCl, and almond flour were used as the raw materials in patty production. Beef meat (mixture of *M. semimembranosus*, *M. semitendinosus*, *M. biceps femoris*; 74.82% moisture, 21.14% protein, 2.93% fat) and beef fat (14.97% moisture, 2.98% protein, and 81.80% fat) obtained from middle‐aged beef carcasses that have completed the rigor‐mortis stage were purchased from a local butcher shop. Almond flour (57.4% fat, 22.5% protein, 4.75% moisture, 9.7% fiber) obtained by grinding after peeling was purchased from a commercial company (Wefood). After beef meat and beef fat were minced separately through a 3‐mm plate with a grinder (AR 160, Arzum) and kept overnight in the refrigerator (4°C), five different groups of patties were produced. In the control group, 80% beef meat, 20% beef fat, and 1.5% NaCl were used. In other groups, beef fat was completely or partially replaced with almond flour (Table 1). All ingredients for each treatment were placed simultaneously in a mixer (AR1129, Arzum) and kneaded for 2 min to obtain a homogeneous dough. Beef patties were shaped (7 cm diameter and 1 cm thickness) with a stainless‐steel ready‐made mold, kept overnight in the refrigerator (4°C) for structural stability, and then cooked with a hot plate (Elektro‐mag, M4060) at 200°C for 8 min (4 min for each surface). Figure 1 shows the physical appearance of the raw and cooked beef patties. The physicochemical properties were determined in raw and cooked samples. However, textural and sensory properties and fatty acid compositions were detected only in cooked samples. The cooked samples were kept at room temperature and analyzed after cooling to 25–30°C.

**TABLE 1:** The ratios of animal fat and/or almond flour used in the production of beef patties.

Treatments	Beef fat (%)	Almond flour (%)
T1	100	0
T2	75	25
T3	50	50
T4	25	75
T5	0	100

### 2.2 Physicochemical analyses

pH and moisture content values of the samples were determined by the methods provided by AOAC International (AOAC, 2005). The pH was measured with a pH meter (GLP 22, Crison Instruments, S.A.) calibrated with buffer solutions (pH: 4 and 7) at 25°C. Color intensities of the samples were detected according to the criteria given by CIE (Commission Internationale de l'Eclairage) based on three‐dimensional (L*, a* and b*) color measurements using a colorimeter (CR‐400, Minolta Co) having a two‐standard observer, 8‐mm aperture and diffuse illumination. L* defines the color lightness (ranging from 0 for black to 100 for white), a* indicates the degree of color between red and green (negative values indicate green color and positive values indicate red color), and b* indicates color degree between yellow and blue (negative values indicate blue colors and positive values indicate yellow colors). Color measurement was performed from both surfaces of beef patties. Thiobarbituric acid‐reactive substance (TBARS) analysis was performed according to Lemon (1975) and TBARS values were defined as μmol MDA/kg. Fatty acid compositions of samples were determined by gas chromatography (Perkin Elmer Clarus 500) with an FID detector. The extraction of fats was carried out with the method given by Folch et al. (1957) and methyl esters of fatty acids were prepared by the method specified by Metcalfe and Schmitz (1961). To estimate the amounts of cooking yield and shrinkage, the following calculations were performed:

$$\text{Cooking yield}\;\left(\%\right)=\left(\frac{\text{cooked weight}}{\mathrm{raw}\;\text{weight}}\right)\times 100$$

$$\text{Shrinkge}\;\left(\%\right)=\left(\frac{\left(\mathrm{raw}\;\text{diameter}-\text{cooked diameter}\right)}{\mathrm{raw}\;\text{diameter}}\right)\times 100$$

### 2.3 Texture profile analysis (TPA)

A texture analyzer (CT3 Texture Analyzer, Brookfield Engineering) was used for texture profile analysis (TPA). Cylindrical samples (2 cm diameter and 1 cm thickness) extracted from beef patties cooled to 25–30°C were analyzed at room temperature with two consecutive compression cycles using a 50.8‐mm‐diameter cylindrical probe (TA 25/1000, Brookfield Engineering Laboratories). One millimeter per second pretest speed, 2 mm/s test, and posttest speed, 3‐s recovery time, and 50% target strain were used in the analysis. Texture profiles of the samples (hardness, adhesiveness, springiness, cohesiveness, resilience, and chewiness) were calculated from the force‐time curves.

### 2.4 Sensory analysis

Sensory evaluation was conducted according to the procedures of AMSA (1978) and IFT (1985). Each beef patty was evaluated in terms of color, odor, taste, texture, and general acceptability. Cooked beef patties were cut into quarters and served randomly at a temperature of 25–30°C to the panelists. The beef patties were evaluated based on a 10‐point hedonic scale, where 1 represented “extremely undesirable” and 10 represented “extremely desirable”. The sensory evaluation was carried out by 10 panelists including five males and five females from the food engineering department in duplicate on each sample. The tests were initiated after the panelists were briefed about the scale and were conducted by the panelists in a room with fluorescent lighting. The panelists were instructed to cleanse their mouths between samples using water and bread.

### 2.5 Statistical analysis

Treatments and cooking process were taken as factors in the study, and experiments were conducted according to the completely randomized design. Two measurements were performed in the physicochemical analyses. However, six measurements were performed in the TPA analysis. The analysis of variance was applied to the obtained data (one‐way ANOVA), and differences between means were compared by Duncan's multiple comparison test at the 95% confidence level (*p* < .05) (IBM SPSS Statistics 20). All data were given as mean values ± standard error in the tables and figures.

## 3 RESULTS AND DISCUSSION

### 3.1 Physicochemical properties

The physicochemical properties of raw and cooked beef patties containing almond flour as a fat replacer are given in Table 2. The pH values of the beef patties were significantly affected by the using ratio of animal fat/almond flour and the cooking process (*p* < .01). The pH value of both raw and cooked beef patties increased with the use of almond flour instead of animal fat, and the highest mean values were determined in the T4 and T5 groups, in which the animal fat was replaced with almond flour at the ratios of 75% and 100%, respectively (*p* < .05). Similarly, Rajkumar et al. (2014) reported that the addition of almond flour to nuggets produced from goat meat increased the pH value. It has also been reported that the use of oat bran instead of animal fat in meatball production (Yılmaz & Dağlıoğlu, 2003) and the use of rice bran fiber in reduced‐fat frankfurters increase the pH value (Choi et al., 2015). In a study conducted by Öztürk and Turhan (2020), it was determined that the substitution of animal fat with pumpkin seed kernel flour increased the pH value of meatballs, and it was suggested that this increase was due to the high free fatty acid concentration in beef fat. The authors reported that replacing beef fat with pumpkin seed kernel flour reduces the amount of beef fat in meatball formulations and therefore increases pH values. Similarly, also in this study, where almond flour was used as a fat substitute and the amount of animal fat in the beef patties decreased depending on the substitution rate, the increase in the pH of the beef patties was probably due to the free fatty acid concentration in the animal fat. In addition, the pH values of beef patty samples increased with the cooking process in all treatments (Table 2). This increase in pH was probably due to imidazolium, the basic R group of the amino acid histidine, being exposed because of protein denaturation during the cooking process (Choi et al., 2015). The cooking process caused an increase in the pH of meatballs was also reported in some other studies (Choi et al., 2016; Elbir et al., 2023).

**TABLE 2:** Physicochemical properties of raw and cooked beef patties containing almond flour as a fat replacer.

	T1	T2	T3	T4	T5
pH					
Raw	5.87 ± 0.01^aA^	5.93 ± 0.01^bA^	5.97 ± 0.01^cA^	6.01 ± 0.01^dA^	6.02 ± 0.01^dA^
Cooked	6.05 ± 0.01^aB^	6.14 ± 0.01^bB^	6.18 ± 0.00^cB^	6.21 ± 0.01^dB^	6.22 ± 0.01^dB^
Moisture content (%)					
Raw	62.65 ± 0.30^aB^	62.82 ± 0.91^aB^	62.23 ± 0.15^aB^	62.86 ± 0.40^aB^	61.55 ± 0.12^aB^
Cooked	57.28 ± 0.02^cA^	56.45 ± 0.02^bA^	55.49 ± 0.03^aA^	54.95 ± 0.40^aA^	54.88 ± 0.07^aA^
TBARS (μmol MDA/kg)					
Raw	22.66 ± 0.39^dA^	7.72 ± 0.47^aA^	9.27 ± 0.28^bA^	10.59 ± 0.10^cA^	11.36 ± 0.29^cA^
Cooked	29.88 ± 1.0^cB^	8.63 ± 0.69^aA^	10.63 ± 0.24^abA^	11.91 ± 1.06^bA^	12.64 ± 0.27^bA^
L*					
Raw	39.76 ± 0.89^aA^	39.75 ± 2.93^aA^	43.13 ± 0.18^aB^	43.97 ± 0.49^aB^	45.07 ± 0.63^aB^
Cooked	31.69 ± 1.85^aA^	29.87 ± 0.84^aA^	29.91 ± 1.71^aA^	28.93 ± 016^aA^	30.93 ± 018^aA^
a*					
Raw	12.37 ± 0.18^aB^	17.65 ± 0.43^aB^	17.74 ± 1.14^aB^	16.54 ± 2.30^aB^	16.47 ± 0.78^aB^
Cooked	6.21 ± 0.27^aA^	6.15 ± 0.01^aA^	6.20 ± 0.21^aA^	6.62 ± 0.07^aA^	6.42 ± 0.33^aA^
b*					
Raw	7.88 ± 0.44^aA^	11.45 ± 0.56^bB^	12.35 ± 0.84^bB^	12.40 ± 1.14^bB^	13.47 ± 0.48^bB^
Cooked	9.48 ± 0.08^bA^	8.22 ± 0.45^aA^	8.18 ± 0.17^aA^	8.32 ± 0.30^aA^	8.41 ± 0.23^aA^
Cooking yield (%)	68.48 ± 0.01^a^	77.75 ± 0.08^b^	85.03 ± 0.33^c^	87.17 ± 0.51^d^	90.81 ± 0.39^e^
Shrinkage	18.75 ± 0.69^d^	14.59 ± 0.69^c^	11.81 ± 0.69^b^	9.03 ± 0.69^a^	7.63 ± 0.69^a^

*Note*: ±: standard error; T1: 100% animal fat, T2: 75% animal fat + 25% almond flour, T3: 50% animal fat + 50% almond flour, T4: 25% animal fat + 75% almond flour, T5: 100% almond flour.

^a–e^: Means marked with different letters in the same line are statistically different from each other (*p* < .05).

^A–B^: Means marked with different letters for raw and cooked samples are statistically different from each other (*p* < .05).

The effect of animal fat substitution on the moisture content of raw beef patties was not significant (*p* > .05). However, the moisture contents in the cooked samples, ranging from 54.88% to 57.28%, were affected by the animal fat replacement significantly (*p* < .01). Moisture content in cooked samples decreased by substituting animal fat with almond flour (*p* < .05), but no significant change was observed after the 50% substitution level (*p* > .05). The decrease in moisture content of beef patties formulated with almond flour was probably the result of an increase in solid content due to almond flour having lower moisture content than animal fat. Some other authors also reported that replacing animal fat with oat bran (Yılmaz & Dağlıoğlu, 2003), chicory root powder (El Zeny et al., 2019), and pumpkin seed flour (Öztürk & Turhan, 2020) reduced the moisture content of beef patties. In addition, the cooking process was also effective on the moisture contents of the samples, and lower mean values were determined for all treatments in the cooked samples (Table 2). This was probably due to moisture loss caused by evaporation during the cooking process. A similar explanation was also reported by Sheridan and Shilton (2002) for moisture loss in beef burger patties during cooking. The use of almond flour as an animal fat replacer in the production of beef patties had a significant effect on the cooking yield and shrinkage (*p* < .01). As the amount of almond flour used in production increased, the cooking yield increased, and the shrinkage decreased (Table 2). While the lowest cooking yield was determined in the control (T1), the highest was detected in the T5 group (*p* < .05). The lower cooking yield in control beef patties was probably due to the excessive fat separation and water release during cooking. The increase in cooking yield with fat substitution might be explained by the low fat in the samples and dietary fiber in almond flour. It was also reported that dietary fibers increased cooking yield and reduced shrinkage in meat products providing a high ability to keep moisture and fat in the meat matrix (Lopez‐Vargas et al., 2014; Selani et al., 2016). In addition, the replacement of animal fat with almond flour contributed to the increase in cooking yield by increasing the solid content of beef patties. Shrinkage of the beef patties improved with increasing levels of almond flour compared to the control, and the lowest shrinkage was determined in the T4 and T5 groups (Table 2). Shrinkage in beef patties is caused by muscle protein denaturation and partly by melted fat and water evaporation during heating (Alakali et al., 2010). The lower shrinkage observed in beef patties with almond flour pointed out the binding and stabilizing effect of almond flour, which restricted changes in the product shape. These cooking yield and shrinkage results agree with other works on beef patties containing different fat replacers (El Zeny et al., 2019; Gök et al., 2011).

Substitution of animal fat with almond flour did not affect the L* and a* values (*p* > .05) while causing a significant change in the b* parameter (*p* < .05) in both raw and cooked beef patties. The b* value of the beef patties using almond flour was higher in the raw samples and lower in the cooked ones than the control (Table 2). Almond flour as a fat replacer caused more yellowness in the raw beef patties, possibly due to the high concentration of yellow pigment. The lower yellowness values of beef patties containing almond flour in cooked samples compared to the control group may be due to the roasting of almond flour result of the cooking process. On the other hand, the cooking process caused decreases in L*, a*, and b* values (Table 2). The decreases in color values during the cooking process can be attributed to increased metmyoglobin concentration or myoglobin denaturation and roasting of almond flour. Similar results were also reported by Gök et al. (2011) and Gao et al. (2022) for meat patties produced with different fat replacers.

The TBARS test has been widely used to measure lipid oxidation in meat and meat products. In this study, TBARS analysis was performed to determine the effect of both fat substitution and the cooking process on lipid oxidation. As can be seen in Table 2, the replacement of animal fat with almond flour was significantly effective on the TBARS values of raw and cooked samples (*p* < .01), and significant differences were detected between the mean values (*p* < .05). Lower TBARS values were obtained in beef patties containing almond flour compared to the control for raw and cooked samples. This effect could be due to the natural antioxidants contained in almond flour. Indeed, it was reported that almonds contain a variety of antioxidant phytochemicals, including phenolic compounds and α‐tocopherol (Colic et al., 2019; Kamil & Chen, 2012; Martins et al., 2017). On the other hand, in samples containing almond flour, the TBARS value increased with the increasing substitution rate. This was probably due to the unsaturated fatty acids of almond flour. However, there was no significant difference between T4 and T5 groups for raw and cooked samples. Similar results were also reported by other authors that the substitution of animal fat with some other substituents causes a decrease in the TBARS value in meatballs (Gao et al., 2022; Noumo et al., 2016; Paula et al., 2019; Poyato et al., 2015). In addition, the cooking process affected the TBARS value and caused increases (Table 2). Some researchers reported that the cooking process affects lipid oxidation in meat products and accelerates oxidative reactions (Jo et al., 2003; Paula et al., 2019; Ramirez et al., 2005). It is evaluated that this increase is related to the release of iron in meat proteins during the cooking process and the damage to the meat's cellular structure by the cooking process (Ramirez et al., 2005; Rojas & Brewer, 2007). In this study, while the increase in TBARS was found to be statistically significant in the control group (*p* < .05), it was not significant in the other groups (*p* > .05). This is probably because, as mentioned above, almond flour contains antioxidant substances. It was also reported in some other studies that the TBARS value of beef patties increased because of the cooking process (Dias et al., 2021; Elbir et al., 2023; Poyato et al., 2015).

Considering the effects of animal fat, high in meat products, on human health, new strategies are being developed to produce healthier meat products by substituting animal fat (Rajkumar et al., 2014). One of these strategies may be using almond flour containing mono‐ and polyunsaturated fatty acids in meat products as an animal fat replacer. In this context, the fatty acid profiles of beef patties produced in different formulations are given in Table 3. Replacing animal fat with almond flour caused differences in the fatty acid profile of beef patties. The total saturated fatty acids (SFA) significantly decreased with the use of almond flour, especially due to the decrease in palmitic acid (C16:0) (*p* < .01). Although there was no significant change in the total monounsaturated fatty acids (MUFA), the oleic acid (C18:1) amount in the samples increased significantly (*p* < .01) with the use of almond flour, and the highest oleic acid amount was determined in the T5 group. This could be mainly due to the predominant oleic acid content of the almond flour. This result indicates that the nutritional and health values of beef patties are enriched as a result of using almond flour instead of animal fat. There were significant differences in the polyunsaturated fatty acids (PUFAs) among the groups (*p* < .01). The PUFAs were increased with the increasing levels of almond flour, and the highest amount of PUFAs was determined in the T5 group. This was probably due to the linoleic acid content of the almond flour. Already, a similar increase was detected in the amount of linoleic acid (C18:2) in the samples. However, the α‐linolenic acid (C18:3) amount decreased significantly with the increasing levels of almond flour. As a result, replacing animal fat with almond flour decreased the SFAs and increased the amounts of oleic and linoleic acids in beef patties. This is important for obtaining healthier products in terms of fatty acid profile. Rajkumar et al. (2014) found similar results in goat meat nuggets incorporated with almond powder and reported that the addition of almonds to goat meat nuggets resulted in a noticeable improvement in the nutritional profile. In another study, the substitution of animal fat with pumpkin seed flour decreased SFAs and increased PUFAs in beef patties (Öztürk & Turhan, 2020).

**TABLE 3:** Fatty acid profiles of raw and cooked beef patties containing almond flour as a fat replacer (% of total fatty acids).

	T1	T2	T3	T4	T5	Significance
C13:0 Tridecanoic acid	0.09 ± 0.01^c^	0.05 ± 0.01^b^	0.05 ± 0.01^b^	0.03 ± 0.01^ab^	0.02 ± 0.01^a^	*
C14:0 Myristic acid	3.23 ± 0.22^a^	2.14 ± 0.21^a^	2.65 ± 0.60^a^	1.61 ± 0.20^a^	2.31 ± 0.25^a^	ns
C15:0 Pentadecanoic acid	0.70 ± 0.63^a^	0.53 ± 0.26^a^	0.35 ± 0.06^a^	0.48 ± 0.16^a^	0.21 ± 0.09^a^	ns
C16:0 Palmitic acid	28.31 ± 1.36^e^	22.51 ± 0.07^d^	18.94 ± 0.40^c^	13.63 ± 0.31^b^	8.60 ± 0.17^a^	**
C18:0 Stearic acid	21.09 ± 1.95^a^	19.32 ± 1.20^a^	20.07 ± 3.51^a^	16.87 ± 1.69^a^	10.92 ± 1.60^a^	ns
ƩSFA	53.43 ± 0.20^d^	44.55 ± 0.66^c^	42.06 ± 2.55^c^	32.61 ± 1.24^b^	22.06 ± 1.10^a^	**
C14:1 Myristoleic acid	1.83 ± 0.51^a^	1.17 ± 0.33^a^	0.85 ± 0.23^a^	0.39 ± 0.19^a^	0.05 ± 0.01^a^	ns
C16:1 (n‐7) Palmitoleic acid	3.47 ± 0.26^c^	2.83 ± 0.41^bc^	1.96 ± 0.65^ab^	1.63 ± 0.08^ab^	0.77 ± 0.05^a^	*
C18:1 (n‐9c) Oleic acid	36.73 ± 0.29^a^	41.39 ± 0.55^ab^	41.00 ± 2.58^ab^	45.41 ± 1.31^b^	51.77 ± 1.47^c^	**
ƩMUFA	42.03 ± 0.49^a^	45.39 ± 1.28^a^	43.80 ± 2.99^a^	47.43 ± 1.57^a^	52.59 ± 1.53^a^	ns
C18:2 (n‐6c) Linoleic acid	3.02 ± 0.11^a^	8.69 ± 0.69^b^	13.03 ± 0.64^c^	19.03 ± 0.51^d^	24.55 ± 0.62^e^	**
C18:3 (n‐6) ɣ‐Linolenic acid	0.25 ± 0.03^b^	0.17 ± 0.03^ab^	0.18 ± 0.01^ab^	0.13 ± 0.01^a^	0.11 ± 0.01^a^	*
C18:3 (n‐3) α‐Linolenic acid	0.36 ± 0.01^e^	0.30 ± 0.01^d^	0.23 ± 0.01^c^	0.13 ± 0.01^b^	0.04 ± 0.01^a^	**
C20:4 (n‐6) Arachidonic acid	0.45 ± 0.01^a^	0.37 ± 0.01^a^	0.36 ± 0.07^a^	0.37 ± 0.05^a^	0.33 ± 0.01^a^	ns
C20:5 (n‐3) EPA	0.29 ± 0.09^a^	0.26 ± 0.05^a^	0.20 ± 0.08^a^	0.14 ± 0.05^a^	0.19 ± 0.11^a^	ns
C22:6 (n‐3) DHA	0.17 ± 0.07^a^	0.28 ± 0.03^a^	0.16 ± 0.03^a^	0.16 ± 0.07^a^	0.14 ± 0.07^a^	ns
ƩPUFA	4.54 ± 0.29^a^	10.06 ± 0.63^b^	14.15 ± 0.45^c^	19.96 ± 0.32^d^	25.36 ± 0.44^e^	**

*Note*: ±: standard error; T1: 100% animal fat, T2: 75% animal fat + 25% almond flour, T3: 50% animal fat + 50% almond flour, T4: 25% animal fat + 75% almond flour, T5: 100% almond flour.

^a–e^: Means marked with different letters in the same line are statistically different from each other (*p* < 0.05).

**: *p* < 0.01; *: *p* < 0.05; ns: not significant.

### 3.2 Textural properties

Textural properties of beef patties with different formulations are given in Table 4. The hardness, resilience, cohesiveness, and chewiness of the samples were significantly affected by the replacement of animal fat with almond flour (*p* < .01), while adhesiveness and springiness were not (*p* > .05). Using almond flour increased the hardness and chewiness compared to the control; the highest values for these parameters were obtained in the group containing 100% almond flour instead of animal fat (T5). Textural properties of meat products strongly depend on the protein, fat, and moisture contents as well as the presence of nonmeat ingredients. In this study, it is thought that decreasing fat, increasing protein, and lower moisture contents due to fat replacement with almond flour caused higher hardness and chewiness in beef patties. In addition, the dietary fiber content of almond flour might be contributed to high hardness and chewiness. Dietary fibers are reported to increase the consistency of meat products by modifying the rheological properties (Cofrades et al., 2008). On the other hand, using almond flour in the beef patties caused a reduction in resilience and cohesiveness, and the lowest values were again determined in the T5 group. This was probably due to the weakening of the bonds among the components in the beef patties because of substituting animal fat with almond flour. Similar textural results were also reported for low‐fat beef burgers and sausages using different fat replacers (Almeida et al., 2014; Tabarestani & Tehrani, 2012). Aslinah et al. (2018) also determined that using adzuki bean flour as a fat replacer in reduced‐fat beef meatballs at high levels caused higher hardness and chewiness, but cohesiveness and springiness were not changed. In another study, Mumyapan et al. (2021) found lower resilience and cohesiveness in bologna‐type sausages produced with different levels of pumpkin seed flour.

**TABLE 4:** Textural properties of raw and cooked beef patties containing almond flour as a fat replacer.

	T1	T2	T3	T4	T5	Significance
Hardness (*N*)	56.54 ± 6.73^a^	91.44 ± 4.24^b^	101.89 ± 2.78^b^	104.37 ± 1.91^b^	177.56 ± 14.76^c^	**
Adhesiveness (mJ)	0.03 ± 0.02^a^	0.05 ± 0.02^a^	0.03 ± 0.02^a^	0.12 ± 0.05^a^	0.12 ± 0.05^a^	ns
Springiness (mm)	6.87 ± 0.36^a^	7.03 ± 0.15^a^	7.17 ± 0.11^a^	7.05 ± 0.07^a^	6.73 ± 0.08^a^	ns
Cohesiveness	0.49 ± 0.01^d^	0.41 ± 0.01^c^	0.37 ± 0.01^b^	0.37 ± 0.01^b^	0.34 ± 0.00^a^	**
Resilience	0.14 ± 0.01^d^	0.11 ± 0.01^c^	0.08 ± 0.00^b^	0.08 ± 0.00^b^	0.05 ± 0.01^a^	**
Chewiness (mJ)	187.02 ± 20.91^a^	262.50 ± 13.16^b^	267.48 ± 10.90^b^	276.62 ± 2.58^b^	408.62 ± 29.25^c^	**

*Note*: ±: standard error; T1: 100% animal fat, T2: 75% animal fat + 25% almond flour, T3: 50% animal fat + 50% almond flour, T4: 25% animal fat + 75% almond flour, T5: 100% almond flour.

^a–c^: Means marked with different letters in the same line are statistically different from each other (*p* < .05).

**: *p* < .01; ns: not significant.

### 3.3 Sensory properties

The sensory properties of beef patties produced with almond flour as a fat replacer are shown in Table 5. There were no significant differences among the groups for all the sensory parameters (*p* > .05). This remarkable result showed that using almond flour in manufacturing beef patties as a fat replacer did not cause a significant change in consumer demand. A similar result was also reported by Rajkumar et al. (2014) in goat meat nuggets containing almond powder in different ratios. Although most studies considered fat replacement with different replacers have succeeded in producing healthier products, some problems in sensory quality have been widely reported (Domínguez et al., 2022; Faria et al., 2015). In this context, almond flour can be considered a potential fat replacer in terms of sensory quality in beef patty production.

**TABLE 5:** Sensory properties of raw and cooked beef patties containing almond flour as a fat replacer.

	T1	T2	T3	T4	T5	Significance
Color	6.95 ± 0.34^a^	7.25 ± 0.30^a^	7.45 ± 0.31^a^	7.25 ± 0.27^a^	7.25 ± 0.33^a^	ns
Odor	7.40 ± 0.29^a^	6.85 ± 0.34^a^	7.85 ± 0.17^a^	7.30 ± 0.31^a^	7.30 ± 0.28^a^	ns
Taste	6.60 ± 0.36^a^	6.45 ± 0.34^a^	7.35 ± 0.25^a^	6.60 ± 0.34^a^	6.80 ± 0.39^a^	ns
Texture	6.50 ± 0.39^a^	7.05 ± 0.32^a^	7.15 ± 0.32^a^	6.70 ± 0.35^a^	6.90 ± 0.41^a^	ns
General acceptability	6.70 ± 0.37^a^	7.00 ± 0.31^a^	7.60 ± 0.21^a^	6.80 ± 0.31^a^	7.20 ± 0.37^a^	ns

*Note*: ±: standard error; T1: 100% animal fat, T2: 75% animal fat + 25% almond flour, T3: 50% animal fat + 50% almond flour, T4: 25% animal fat + 75% almond flour, T5: 100% almond flour.

Means marked with same letters in the same line are not statistically different from each other (*p* > .05).

Abbreviation: ns, not significant.

## 4 CONCLUSIONS

Replacing animal fat with almond flour caused considerable changes in the physicochemical and textural properties of beef patties. Especially the reductions in TBARS and shrinkage and the increase in cooking yield and color properties are notable in terms of product quality. Considering the fatty acid profile, the use of almond flour resulted in significant improvement in fatty acids such as C16:0, C18:1, and C18:2. The instrumental texture properties of hardness, cohesiveness, resilience, and chewiness changed significantly with the replacement of almond flour. On the other hand, the sensory properties of beef patties with almond flour were similar to the control. In conclusion, the replacement of animal fat with almond flour in beef patties resulted in appreciable improvements in the product quality, such as nutritional profile, and could be a promising approach for the development of functional beef patties. However, it is thought that there is a need for new studies examining the changes that will occur during storage under different conditions.

## AUTHOR CONTRIBUTIONS

**Mine Kirkyol:** Conceptualization (equal); formal analysis (equal); investigation (equal); methodology (equal); validation (equal); visualization (equal); writing – original draft (equal); writing – review and editing (equal). **Ahmet Akköse:** Conceptualization (equal); investigation (equal); methodology (equal); supervision (equal); writing – original draft (equal); writing – review and editing (equal).

## FUNDING INFORMATION

This research did not receive any specific grant from funding agencies in the public, commercial, or not‐for‐profit sectors.

## CONFLICT OF INTEREST STATEMENT

The authors declare that they do not have any conflict of interest.

## Data Availability

The data that support the findings of this study are available from the corresponding author upon reasonable request.
